# Molecular characteristics and pathogenicity-associated phenotypes of methicillin-resistant *Staphylococcus aureus* sequence type 398 clinical isolates from a women and children’s hospital in Southwest China

**DOI:** 10.3389/fpubh.2026.1882026

**Published:** 2026-07-06

**Authors:** Li Liu, Ziyi Yan, Jie Chen, Yunhan Fu, Wei Zhou, Xingxin Liu, Yingying Li, Jiaji Ling, Yali Cui, Linghan Kuang, Yongmei Jiang

**Affiliations:** 1 Department of Laboratory Medicine, West China Second University Hospital, Sichuan University, Chengdu, China; 2 Department of Blood Transfusion, Beijing Anzhen Nanchong Hospital of Capital Medical University & Nan-chong Central Hospital, Nanchong, China; 3 Key Laboratory of Birth Defects and Related Diseases of Women and Children of Ministry of Education, West China Second University Hospital, Sichuan University, Chengdu, China; 4 College of Computer Science, Sichuan University, Chengdu, China; 5 West China School of Medicine, West China Hospital, Sichuan University, Chengdu, China; 6 Department of Laboratory Medicine, Tibet Autonomous Region Women's and Children's Hospital, Lhasa, China; 7 Department of Laboratory Medicine, Meishan Women and Children’s Hospital, Alliance Hospital of West China Second University Hospital, Sichuan University, Meishan, Sichuan, China; 8 Department of Laboratory Medicine, West China Second University Hospital (Tianfu), Sichuan University (Sichuan Provincial Children’s Hospital), Meishan, Sichuan, China; 9 Department of Laboratory Medicine, Chengdu Hi-Tech Zone Hospital for Women and Children (Chengdu Hi-Tech Zone Hospital for Maternal and Child Healthcare), Chengdu, China

**Keywords:** antimicrobial resistance, China, MRSA, ST398, *Staphylococcus aureus*, whole-genome sequencing

## Abstract

**Background:**

Methicillin-resistant *Staphylococcus aureus* (MRSA) sequence type 398 (ST398), initially recognized as a livestock-associated lineage, has been increasingly reported in human infections. However, the molecular characteristics, antimicrobial resistance profiles, phylogenetic context, and pathogenicity-associated phenotypes of clinical MRSA ST398 isolates in China remain insufficiently defined. Here, we characterized such isolates from Southwest China using genomic, antimicrobial susceptibility, and phenotypic analyses.

**Methods:**

From January 2020 to June 2023, 23 nonduplicate MRSA ST398 isolates were identified among 305 nonduplicate clinical isolates from West China Second University Hospital. Whole-genome sequencing was performed for molecular typing, antimicrobial resistance determinant analysis, phylogenetic analysis, and virulence-associated gene screening. Growth, biofilm formation, cell adhesion, hemolysis, mouse skin abscess, and *Galleria mellonella* survival assays of selected clinical isolates within each lineage were performed to evaluate pathogenicity-associated phenotypes.

**Results:**

ST398-V-t011 was the dominant lineage in 15/23 of the isolates (65.2%). *SCCmec* type V and spa type t011 were detected in 22/23 (95.7%) and 16/23 (69.6%) of the isolates, respectively. All isolates were *mecA*-positive and were phenotypically resistant to penicillin G and oxacillin, but remained susceptible to vancomycin and linezolid. Erythromycin resistance and clindamycin resistance, mainly associated with *ermC*, were each detected in 15/23 of the isolates (65.2%). Phylogenetic analysis showed that 22/23 local isolates clustered within a China-associated clade, and LSD2-based dating estimated that the internal node of this clade was approximately 2008, providing temporal context for the observed clustering pattern. Virulence-associated gene screening revealed the conservation of many genes across isolates, whereas several accessory genes varied. Lineage-based phenotypic assays using selected isolates revealed heterogeneous pathogenicity-associated readouts: ST398-V-t2383 and ST398-V-t1580 displayed relatively higher biofilm formation and/or cell adhesion capacities, whereas ST398-V-t011 showed higher hemolytic activity and higher pathogenicity-associated readouts in mouse skin abscess and *G. mellonella* models.

**Conclusion:**

The obtained clinical MRSA ST398 isolates predominantly belonged to the ST398-V-t011 lineage and had a *mecA*-positive beta-lactam-resistant phenotype with variable non-beta-lactam resistance. Although a China-associated genomic cluster was identified, this finding should not be interpreted as direct evidence of the transmission direction or a definite transmission chain. The observed phenotypic heterogeneity provides exploratory pathogenicity-associated data and supports continued genomic surveillance and multicenter validation of clinical MRSA ST398 in China.

## 1 Introduction

*Staphylococcus aureus* (*S. aureus*) is a gram-positive, facultative anaerobic bacterium that colonizes the skin and nasal passages of approximately 30% of the human population (1). While *S. aureus* often coexists asymptomatically with its host, this bacterium can cause a wide spectrum of diseases, ranging from mild skin and soft-tissue infections, such as impetigo, folliculitis, and boils, to life-threatening conditions, such as pneumonia, endocarditis, sepsis, and toxic shock syndrome (2, 3). Methicillin-resistant *S. aureus* (MRSA) represents a clinically important resistant phenotype of *S. aureus* and has been considered by some researchers to be a distinct group of pathogens because of its epidemiological and therapeutic significance (4). MRSA is distinguished from other *S. aureus* strains by its resistance to *β*-lactam antibiotics, including methicillin, oxacillin, and other penicillin-derived drugs. This resistance is conferred mainly by the acquisition of the *mecA* gene, which encodes penicillin-binding protein 2a (PBP2a). PBP2a has a low affinity for β-lactam antibiotics and allows the bacteria to continue synthesizing cell walls even in the presence of these drugs (5, 6).

MRSA has emerged as a substantial global health threat. In the United States, from 2012 to 2017, the unadjusted mortality rates of MRSA were estimated to be 29% for patients with hospital-onset bloodstream infections and 18% for patients with community-onset bloodstream infections (7). In Europe, the all-cause mortality rate 14 d after the first isolation of MRSA was 20.8% for patients with invasive infections (8). In India, a meta-analysis focusing on MRSA reported a pooled proportion of MRSA infection of 26.8% among patients with *S. aureus* infection (9). MRSA infections lead to increased morbidity and mortality rates, extended hospital stays, and increased healthcare resource utilization (10).

Among the diverse MRSA lineages, sequence type 398 (ST398) has attracted considerable attention. Initially, ST398 was identified as a livestock-associated (LA) MRSA clone, primarily colonizing pigs, cattle, and other farm animals (11, 12). However, subsequent studies have shown that ST398 is not restricted to livestock-associated settings and has been detected to colonize humans and cause clinical infections worldwide (13–15). In Europe, ST398 was reported among swine farmers in the Netherlands and was subsequently identified in livestock-related environments and humans with livestock contact (16, 17). In North America, ST398 has also been documented in both livestock and human populations (18, 19). These findings indicate that the host-associated and ecological background of ST398 is complex. Nevertheless, compared with those of livestock-associated ST398 and methicillin-susceptible *S. aureus* CC398, the molecular characteristics, antimicrobial resistance profiles, phylogenetic context, and pathogenicity-associated phenotypes of clinical MRSA ST398 isolates in China remain insufficiently defined.

In this study, we investigated clinical MRSA ST398 isolates collected from a women and children’s hospital in Southwest China. We combined whole-genome sequencing, molecular typing, antimicrobial susceptibility testing, resistance determinant analysis, virulence-associated gene screening, and phylogenetic analysis to characterize the genomic and clinical microbiological features of these isolates. In addition, lineage-based phenotypic assays using selected clinical isolates were performed to evaluate growth, biofilm formation, cell adhesion, hemolysis, mouse skin abscess formation, and *Galleria mellonella* larval survival under defined experimental conditions. Rather than inferring a definite transmission route or lineage-wide virulence mechanism, this study aimed to provide regional genomic, antimicrobial resistance, phylogenetic, and exploratory pathogenicity-associated data for clinical MRSA ST398 isolates in Southwest China.

## 2 Materials and methods

### 2.1 *Staphylococcus aureus* strain collection, clinical relevance, and identification

**Figure 1:**
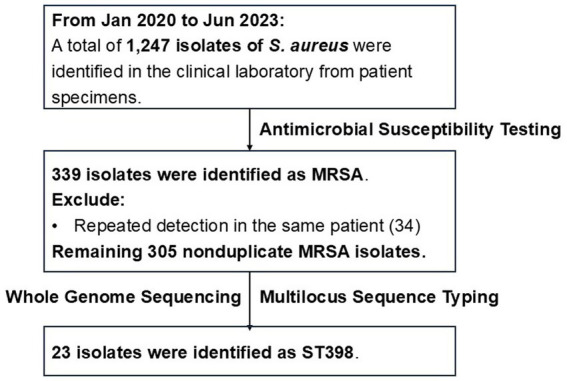
Workflow for the inclusion and exclusion of clinical *S. aureus* isolates and identification of MRSA ST398 isolates.

The collection of *S. aureus* isolates began in January 2020 and ended in June 2023. These isolates were obtained from clinical specimens collected from patients who had been admitted to West China Second University Hospital, Sichuan University, and who had long-term residence in Southwest China. This hospital is a national regional medical center in Southwest China and one of the largest specialized hospitals for women and children in China. During the study period, a total of 1,247 *S. aureus* isolates were detected from clinical samples. Among them, 339 isolates were identified as MRSA. After repeated isolates from the same patients were excluded, 305 nonduplicate MRSA isolates remained. Only the first isolate from each patient was retained. After whole-genome sequencing and multilocus sequence typing, 23 nonduplicate MRSA isolates were identified as ST398 and included in this study (Figure 1).

The clinical records of all the patients were reviewed. All included patients had been diagnosed by the treating physicians with *S. aureus*-associated diseases according to the clinical manifestations, imaging findings, laboratory test results, microbiological culture results, and corresponding ICD diagnostic criteria. The clinical relevance of each isolate was therefore determined based on both microbiological evidence and the clinical diagnosis rather than microbiological culture results alone. Most of the respiratory samples were sputum samples, and isolates were included only when the patients had compatible clinical and laboratory findings and were diagnosed by the treating physicians as having *S. aureus*-related respiratory infection. Active colonization screening samples were not included.

The hospital’s clinical laboratory has been accredited by the College of American Pathologists (CAP) and the ISO15189 accreditation standard. Specimens were collected by specialized physicians or nurses according to the Standard Operating Procedure (SOP) of ISO/TS 20658:2017, Medical laboratories—Requirements for collection, transport, receipt, and handling of samples. The isolates were cultured on Columbia blood agar plates (CBA; Autobio, Zhengzhou, China) and incubated at 35 °C for 24–48 h in a 5% carbon dioxide (CO_2_) environment. All the isolates were identified as *S. aureus* by matrix-assisted laser desorption ionization time-of-flight mass spectrometry (MALDI-TOF MS; Vitek MS system; bioMérieux, Rhône, France).

### 2.2 Antimicrobial susceptibility testing and resistance determinant analysis

Antimicrobial susceptibility testing was performed using VITEK AST-GP67 cards (bioMérieux, Rhône, France) according to the manufacturer’s instructions. The antimicrobial agents tested included penicillin G, oxacillin, erythromycin, clindamycin, gentamicin, rifampin, moxifloxacin, ciprofloxacin, trimethoprim–sulfamethoxazole, tetracycline, nitrofurantoin, linezolid, vancomycin, quinupristin/dalfopristin, and tigecycline. Cefoxitin screening and inducible clindamycin resistance testing were also performed. *S. aureus* ATCC 25923 was used as the quality control strain. Antimicrobial susceptibility results were interpreted according to CLSI M100 criteria.

Genome-based antimicrobial resistance determinants were identified from assembled genomes using the AMRsearch/PAARSNP antimicrobial resistance genotyping and phenotype-inference pipeline implemented in Pathogenwatch (20, 21). This pipeline detects resistance genes and resistance-associated mutations by comparing assembled genome sequences against species-specific curated antimicrobial resistance libraries.^1^ For *S. aureus*, the curated AMR library is based on the NCBI taxonomy code 1280; in this analysis, the PAARSNP AMR library version was 0.0.16. The library defines antimicrobial resistance markers, including the presence/absence of genes and resistance-associated nucleotide or amino acid substitutions, together with the sequence identity and coverage thresholds required for calling each determinant. The resulting genotype-based resistance determinants and inferred resistance profiles were summarized for each isolate. Phenotypic antimicrobial susceptibility testing was used as the primary basis for antimicrobial susceptibility categorization, whereas the genome-based AMR analysis was used to identify corresponding resistance determinants.

### 2.3 Genome sequencing, molecular typing, and virulence-associated gene screening

Genome sequencing and the identification of *S. aureus* strains were performed as previously reported (22). Briefly, the genome data were assembled with SPAdes software (v3.14.1) (23) and then annotated with Prokka software (v1.14.5) (24), and the sequence types (STs) of the strains were identified using mlst software (v2.19.0) via a comparison with the multilocus sequence typing (MLST) database (25). Assembly quality was evaluated using quality control metrics exported from Pathogenwatch.^2^ In Pathogenwatch, species are assigned using the Speciator tool, which compares genome assemblies with a curated reference library using Mash-based distances (26). Assembly quality was assessed using the Pathogenwatch QC status together with genome length, number of contigs, largest contig length, N50, GC content, number of ambiguous bases per 100 kbp, and average contig length. Assemblies were considered acceptable when they passed the Pathogenwatch QC assessment, were assigned as *Staphylococcus aureus*, and showed assembly metrics consistent with those of *S. aureus* genomes without evidence of substantial ambiguous bases or potential mixed-species contamination. The assembly quality metrics for the 23 MRSA ST398 isolates are provided in Supplementary Table S1.

The *Staphylococcus* protein A gene (*spa*) was typed using the Center for Genomic Epidemiology website^3^ (27), and staphylococcal cassette chromosome *mec* (*SCCmec*) was typed using *SCCmec*Finder (28). *S. aureus* capsular polysaccharide types were identified based on the sequences of the cap locus, and the corresponding type was identified when the *capH* and *capK* gene sequences in the target genome were > 95% identical to the reference sequences (CP5: GenBank CP000253.1; CP8: GenBank U73374.1; https://www.ncbi.nlm.nih.gov/nuccore). Virulence-associated genes were evaluated using ABRicate software (v1.0.1) (Seemann T, Abricate, GitHub https://github.com/tseemann/abricate) based on the VFDB database and GenBank (22, 29, 30).

### 2.4 Phylogenetic analysis

We searched and filtered *S. aureus* records from the PubMLST database^4^ to describe the phylogenetic context of ST398 isolates from Southwest China. As of September 2024, a total of 42,958 *S. aureus* records were available in the PubMLST database. Among them, 1763 records were identified as ST398, and 1,164 PubMLST ST398 genomes remained after records without available genome sequences or sampling years were excluded. Together with the 23 local ST398 genomes sequenced in this study and 7 standard/reference genomes, 1,194 genomes were initially included in the phylogenetic analysis and phylogenetic dating.

The genome sequences were analyzed using Roary software (v3.13.0) to create a multiFASTA alignment of core genes (>99%) (31). SNP-site software was subsequently used to extract variable sites and remove duplicate sites from the alignment (32). A maximum-likelihood phylogenetic tree was constructed using IQ-TREE (v2.4.0) with the best-fit nucleotide substitution model selected by ModelFinder (33, 34). The sampling dates were provided as tip-date calibrations using a date file. Year-level sampling dates were used for publicly available PubMLST genomes when only the year was available, whereas exact sampling dates were used for the local isolates sequenced in this study.

**Figure 2:**
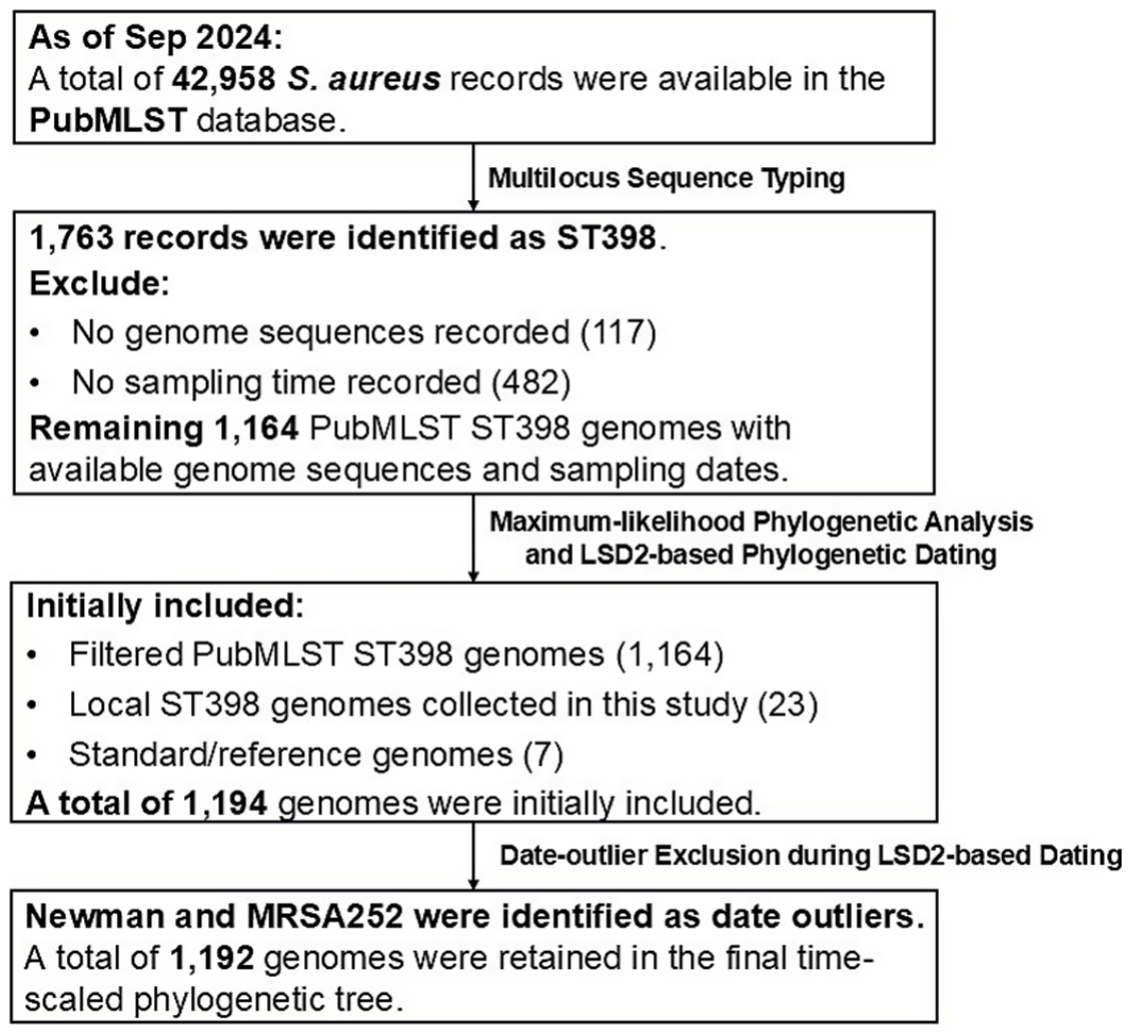
Workflow for the inclusion and exclusion of the genomes used in the phylogenetic analysis and LSD2-based phylogenetic dating.

Phylogenetic dating was performed using the least-squares dating method implemented in LSD2 through IQ-TREE with the command options `--date DATE_FILE --date-outlier 3 --date-ci 100` (35). These settings allowed the detection of date outliers using a Z score threshold of 3 and the estimation of confidence intervals for node dates based on 100 replicates. Newman and MRSA252 were identified as date outliers and were excluded during LSD2-based dating, leaving 1,192 genomes in the final time-scaled phylogenetic tree (Figure 2). The temporal signal was assessed with a root-to-tip regression model using the maximum-likelihood tree and sampling dates. The resulting phylogenetic tree was visualized and annotated using the Interactive Tree of Life (iTOL, v6.9.1) (36).

### 2.5 Selection of isolates for lineage-based phenotypic assays and growth curve assays

For phenotypic assays, lineages were defined according to the combined ST398-*SCCmec*-spa profile, such as ST398-V-t011. Clinical isolates were selected within each lineage according to the number of experimental units required for each assay. When the number of available isolates within a lineage was equal to or greater than the required number of experimental units, the isolates were numbered and randomly selected without replacement whenever possible. For lineages represented by fewer isolates than the number of experimental units, all available isolates were included and reused as evenly as possible across experimental units. For example, both isolates belonging to the ST398-V-t1451 lineage were included when more than two experimental units were required, with approximately balanced allocation across repeated assays, mice, or larvae. For lineages represented by a single isolate, the available isolate was used for all experimental units. Therefore, the experimental units described below represent selected isolate-level measurements when sufficient isolates were available, but repeated assays, animals, or larvae from the same isolate when the lineage contained fewer available isolates. The detailed isolate-allocation scheme for each phenotypic assay is provided in Supplementary Table S2.

Growth curve assays were performed as previously reported, with minor modifications (37). Briefly, a single colony of each *S. aureus* strain was picked and inoculated into 5 mL of tryptic soy broth (TSB; BD Biosciences, NJ, United States), followed by overnight culture with shaking. The overnight culture was then diluted 1:200 into 50 mL of fresh TSB and incubated at 37 °C with shaking at 220 rpm. The OD562 values were measured every hour for 12 h and again at 24 h to monitor bacterial growth. Although OD600 is the most commonly used wavelength for monitoring bacterial growth, optical density measurements can be performed at other visible wavelengths depending on the available instrument configuration and experimental setup (38). Previous studies have used the OD562 to estimate *S. aureus* bacterial concentration or growth under defined experimental conditions (39, 40). In the present study, OD562 was used because the available microplate reader was equipped with a 562-nm detection channel, and all selected isolates were measured using the same wavelength, medium, incubation conditions, and blank correction, allowing the relative comparison of growth patterns among the tested lineages. TSB medium without bacterial inoculation was included as the blank control. The reference strain USA300 was used as a quality-control strain. The assay was performed with three experimental units for each lineage, with isolate allocation performed according to the strategy described above, and all valid values were included in the analysis.

### 2.6 Semiquantitative biofilm formation assay

A semiquantitative biofilm formation assay was performed as previously reported (41). Briefly, overnight *S. aureus* cultures were diluted 1:200 in TSB supplemented with 1.0% glucose (Sigma–Aldrich, MO, USA), dispensed (200 μL) into 96-well microtiter plates (BD Biosciences, NJ, USA) and incubated at 37 °C for 24 h. After the incubation, the bacterial suspension was discarded, and the plates were washed twice with sterile PBS to remove free bacteria from the wells. Then, 200 μL of a methanol solution (Kelong, Sichuan, China) was added to each well and incubated for 15 min to fix the samples, and the samples were stained with a crystal violet solution (Solarbio, Beijing, China) for 15 min. After the samples were washed, 200 μL of a 30% glacial acetic acid solution (Aopusheng, Tianjin, China) was added to each well, the samples were shaken at 300 rpm for 5 min, and the OD_495_ values were measured with a microplate reader (SpectraMax ABS plus, Molecular Devices, Shanghai, China). *Staphylococcus epidermidis* ATCC 12228 and ATCC 35984 were used as a negative control and a positive control, respectively, but were not included in the comparative statistical analysis among the *S. aureus* groups; TSB was used as the blank control. Blank-corrected OD495 values were calculated by subtracting the mean OD495 of the TSB blank wells from each test well. The assay was performed with four experimental units for each lineage, with isolates allocated according to the strategy described above, and the resulting blank-corrected OD495 values were used for the subsequent statistical analysis. The interpretive standards were as follows: (1) OD_495_ value < negative control was defined as negative; (2) negative control < OD_495_ value < 2 × positive control was defined as weakly positive; and (3) OD_495_ value > 2 × positive control was defined as strongly positive.

### 2.7 Cell adhesion assay

Cell adhesion assays were performed as described in a previous study (42). Briefly, A549 lung epithelial cells were grown in DMEM (Gibco, MA, USA) supplemented with 10% fetal bovine serum (AusgeneX, Australia) at 37 °C with 5% CO_2_; *S. aureus* strains were inoculated and incubated for 6 h to reach the logarithmic phase of growth, after which the cells were resuspended in serum-free DMEM. The cells and bacteria were incubated in 12-well plates at 37 °C for 2 h (5 × 10^5^ cells/well; MOI = 10:1). This MOI was selected based on the previously reported *S. aureus* adhesion assay protocol and was intended to provide sufficient bacterial attachment for quantitative CFU recovery while avoiding the excessive disruption of the epithelial cell monolayer during the short incubation period. After the incubation, the plates were washed with sterile PBS, and the cells were digested with trypsin–EDTA (Biosharp, Anhui, China) for 3 min at 37 °C. After the cells were lysed with 0.05% Triton X-100, the bacterial colony forming units (CFUs) were determined by plating serial dilutions of epithelial cell lysates onto Columbia blood agar (CBA) plates. The *S. aureus* strain USA300 was used as the control, and the assay was performed with four experimental units for each lineage, with the isolates allocated according to the strategy described above.

### 2.8 Hemolytic ring assay and hemolytic activity assay

The hemolytic ring assay and hemolytic activity assay were performed as described previously (37). First, hemolysis was assessed by measuring the formation of hemolytic rings on Columbia blood agar (CBA) plates. Briefly, overnight cultures of *S. aureus* bacterial suspensions (10 μL, OD_562_ = 1.0) were added dropwise onto CBA plates, which were subsequently incubated at 37 °C for 24 h and 48 h; then the diameters of the hemolytic rings and colonies were measured. The thickness of the hemolytic ring was determined by calculating the radius of the hemolytic ring minus the radius of the colony, and the assay was performed with three experimental units for each lineage, with the isolates allocated according to the strategy described above. Second, for the hemolytic activity assay, overnight *S. aureus* bacterial suspension cultures were centrifuged at 5500 × g for 2 min at 4 °C, after which the supernatants were filtered through 0.22-μm filters (Millipore, MA, USA). Then, 100 μL of the supernatant was added to 875 μL of PBS (Servicebio, Hubei, China), incubated with 25 μL of defibrinated rabbit blood (Solarbio, Beijing, China) at 37 °C for 1 h, and centrifuged at 6000 × g for 2 min. The OD_562_ values of the supernatants were measured, and defibrinated rabbit blood treated with Triton X-100 (Beyotime, Shanghai, China) or 0.9% NaCl (Kelong, Guangdong, China) was used as a positive control or a negative control, respectively. The hemolytic activity assay was performed with three experimental units for each lineage, with the isolates allocated according to the strategy described above, and the hemolytic activity was calculated as follows: (OD_562_ test value – OD_562_ negative control)/(OD_562_ positive control – OD_562_ negative control).

### 2.9 Mouse skin abscess model

Mouse skin abscess models were established as described previously (37, 41). The bacterial suspension was first adjusted to a 2.0 McFarland turbidity standard. For each selected isolate, the turbidity-adjusted suspension was serially diluted and plated on Columbia blood agar plates to verify the viable bacterial concentration before infection, and the final inoculum was standardized to 6 × 10^8^ CFUs/100 μL.

Four- to six-week-old male BALB/c mice weighing 18–20 g were purchased from Chengdu Dashuo Co., Ltd., and five mice were included in each lineage group, with the selected isolates allocated according to the strategy described above. The mice were anesthetized with 1% (mass/volume) sodium pentobarbital at 50 mg/kg body weight. Then, 100 μL of the *S. aureus* bacterial suspension containing 6 × 10^8^ CFUs was subcutaneously inoculated into the back of each mouse. Sterile PBS (100 μL) was inoculated as a negative control. The abscess area was assessed daily by measuring the maximum length and width of the developing lesion. The lesion area was calculated using the following formula: A = L × W, where L represents length and W represents width. In accordance with the AVMA Guidelines for the Euthanasia of Animals, the mice were euthanized with 1% (mass/volume) sodium pentobarbital at 200 mg/kg body weight.

### 2.10 *Galleria mellonella* larval survival model

*Galleria mellonella* larval survival models were established as described previously (37). Briefly, *S. aureus* cultures in the logarithmic phase of growth were centrifuged, washed, and resuspended in sterile PBS. The bacterial suspension was first adjusted to a 2.0 McFarland turbidity standard. For each selected isolate, the turbidity-adjusted suspension was serially diluted and plated on Columbia blood agar plates to verify the viable bacterial concentration before larval infection, and the final inoculum was standardized to 6 × 10^8^ CFUs/100 μL.

*Galleria mellonella* larvae were purchased from Tianjin Kaideruixin Co., Ltd., and ten larvae were included in each lineage group, with the selected isolates allocated according to the strategy described above. A 100-μL bacterial suspension containing 6 × 10^8^ CFUs was injected between the second and third prolegs of each larva using an insulin needle. Sterile PBS (100 μL) was used as the negative control. The larvae were then placed in a 35 °C incubator, after which the survival time was recorded. Survival curves were generated using GraphPad Prism (version 8.0.1, Boston, MA, USA). Since all infected *Galleria mellonella* larvae died during the observation period, no euthanasia procedure was applied.

### 2.11 Statistical analysis

Statistical analyses were performed using Python (v3.13.4) with relevant scientific-computing libraries and the Statistical Package for the Social Sciences (SPSS, v25.0; Chicago, IL, USA). Continuous variables are presented as the means ± standard deviations (SDs), unless indicated otherwise. For the phenotypic assays, each ST398 lineage was represented by selected clinical isolates according to the number of experimental units required for each assay. When sufficient isolates were available within a lineage, experimental units were assigned to nonduplicate isolates whenever possible. When the number of available isolates was smaller than the number of experimental units, the isolates were reused as evenly as possible.

Clustering analyses were performed as exploratory tools to summarize the phenotypic patterns among the ST398 lineages under the tested experimental conditions. Because the availability of isolates differed among lineages, these analyses were not intended to define stable biological lineages or to support a definitive lineage-wide inference. For the biofilm formation and cell adhesion assays, Ward’s minimum variance hierarchical clustering based on Euclidean distance was used because these assays generated replicate-level continuous measurements for each lineage and allowed grouping according to overall phenotypic similarity. For assays with a single major quantitative endpoint, including hemolytic ring thickness, hemolytic activity, mouse skin abscess area, and the area under the survival curve (AUC) in the *Galleria mellonella* model, k-means clustering was used to classify lineages into low-, intermediate-, or high-phenotype groups. The number of clusters was determined by considering the silhouette coefficient together with the biological interpretability of the phenotype distribution. Where applicable, the consistency of the cluster assignments was checked by comparing the results of the hierarchical clustering and k-means clustering analyses.

Before group comparisons, the normality of residuals and homogeneity of variance were assessed using the Shapiro–Wilk test and Levene’s test, respectively. When the assumptions for ordinary one-way ANOVA were reasonably satisfied, group differences were assessed using one-way ANOVA followed by Tukey’s honestly significant difference (HSD) *post hoc* test. When heterogeneity of variance was detected or cluster sizes were unequal, Welch’s ANOVA followed by the Games–Howell post hoc test was used as a more conservative approach. For the *Galleria mellonella* larval survival assay, survival curves were compared using the log-rank test and Breslow test. In addition, AUC values were calculated to summarize the overall survival profile of each lineage group and were used for exploratory cluster-based comparisons. For repeated experimental measurements, data points were inspected for potential technical errors before statistical analysis. Data exclusion was permitted only when a predefined technical reason was documented, such as contamination, inoculation failure, plating or counting failure, instrument reading error, or sample-processing error. Data points were not excluded solely based on statistical extremeness. No repeated measurements met these predefined exclusion criteria; therefore, all valid experimental data points were included in the final analyses. A two-sided *p* value < 0.05 was considered to indicate statistical significance.

### 2.12 Ethics

The clinical experimental plan was approved by the Clinical Trial Ethics Committee of West China Second University Hospital, Sichuan University (2023–290). The requirement for informed consent to participate was waived by the Clinical Trial Ethics Committee of West China Second University Hospital, Sichuan University, according to the Chinese government “Measures for the Ethical Review of Life Science and Medical Research Involving Human Beings” (Chapter III, Article 32, https://www.gov.cn/zhengce/zhengceku/2023-02/28/content_5743658.htm).

Studies involving animal participants were reviewed and approved by the Animal Ethics Committee of West China Hospital, Sichuan University (20250620001).

This study was conducted in accordance with the Declaration of Helsinki.

## 3 Results

### 3.1 Clinical characteristics of the *Staphylococcus aureus* isolates

A total of 23 nonduplicate MRSA ST398 isolates were collected at West China Second University Hospital from January 2020 to June 2023. The detailed clinical information and demographic characteristics of the patients and isolate sources are summarized in Table 1. Consistent with the clinical setting of a women and children’s hospital, most patients were infants or young children. Among the patients, 56.5% were male and 43.5% were female. The ages ranged from 14 days to 58 years, with a median age of 0.75 years (interquartile range, 0.16–2.58 years). No documented history of livestock contact within 1 month before disease onset was recorded in the available clinical information. The clinical diagnoses included pneumonia, abscess, sepsis, and urinary tract infection (UTI), and the corresponding samples included sputum, alveolar lavage fluid (ALF), tracheal catheter, central venous catheter (CVC), pus, and midstream urine. After treatment, all the patients were cured or experienced a clinical improvement.

**Table 1:** Clinical characteristics and sources of specimens from 23 patients infected with MRSA ST398 isolates.

Characteristics	Patients	%
Sex		
Male	13	56.5
Female	10	43.5
Age		
Median age (P25–P75)	0.75 (0.16–2.58)	
<30 days	2	8.7
30 days–1 year	10	43.5
1 year–4 years	7	30.4
5 years–17 years	3	13.0
≥18 years	1	4.3
Diagnosis		
Pneumonia	19	82.6
Abscess	2	8.7
Sepsis	1	4.3
Urinary tract infection (UTI)	1	4.3
Specimen type		
Sputum	17	73.9
Alveolar lavage fluid (ALF)	1	4.3
Tracheal catheter	1	4.3
Central venous catheter (CVC)	1	4.3
Pus	2	8.7
Midstream urine	1	4.3
Prognosis		
Cured	19	82.6
Improved and/or transferred	4	17.4
Dead	0	0.0
Total	23	100.0

### 3.2 Molecular typing of *Staphylococcus aureus* isolates

All 23 MRSA ST398 genome assemblies passed the Pathogenwatch quality-control assessment and were assigned as *Staphylococcus aureus*. The genome sizes ranged from 2,696,814 to 2,883,000 bp, with GC contents of 32.74–32.87%. The assemblies contained 45–90 contigs, with N50 values ranging from 139,631 to 291,869 bp. No ambiguous bases were detected in any assembly, with 0 Ns per 100 kbp for all the isolates. These assembly quality metrics supported the suitability of the genomes for subsequent molecular typing, the antimicrobial resistance determinant analysis, virulence-associated gene screening, and phylogenetic analysis (Supplementary Table S1).

**Figure 3:**
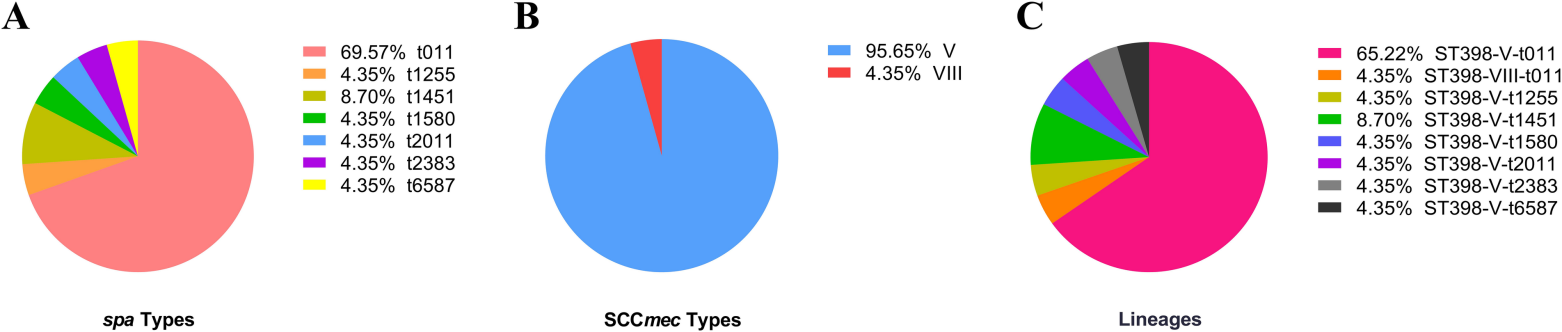
Distributions of **(A)** *spa* types, **(B)** *SCCmec* types, and **(C)** combined molecular lineages among the 23 clinical MRSA ST398 isolates.

A total of 7 *spa* types and 2 *SCCmec* types were detected among the 23 clinical MRSA ST398 isolates (Figure 3). The most common *spa* type was t011, which was identified in 16 isolates (69.57%), followed by t1451 in 2 isolates (8.70%). The remaining *spa* types, including t1255, t1580, t2011, t2383, and t6587, were each identified in 1 isolate (4.35%). *SCCmec* type V was predominant and was detected in 22 isolates (95.65%), whereas *SCCmec* type VIII was detected in 1 isolate (4.35%).

When *spa* type and *SCCmec* type were combined, ST398-V-t011 was the predominant molecular lineage, accounting for 15 isolates (65.22%). The remaining lineages included ST398-V-t1451 in 2 isolates (8.70%) and ST398-VIII-t011, ST398-V-t1255, ST398-V-t1580, ST398-V-t2011, ST398-V-t2383, and ST398-V-t6587 in 1 isolate each (4.35% each) (Figure 3).

### 3.3 Antimicrobial susceptibility and resistance gene profiles

Antimicrobial susceptibility testing was performed for the 23 clinical MRSA ST398 isolates, and the results were interpreted in accordance with the CLSI M100 criteria. All the isolates were resistant to penicillin G and oxacillin, consistent with their MRSA phenotype. In contrast, all the isolates remained susceptible to nitrofurantoin, linezolid, vancomycin, quinupristin/dalfopristin, and tigecycline. Resistance to most non-beta-lactam agents was uncommon, with only 1 isolate each showing resistance to rifampin, moxifloxacin, ciprofloxacin, trimethoprim–sulfamethoxazole, and tetracycline. No isolate was categorized as resistant to gentamicin, although 1 isolate showed intermediate susceptibility. The antimicrobial susceptibility profiles and corresponding resistance determinants are summarized in Table 2.

**Table 2:** Antimicrobial susceptibility and resistance determinant profiles of 23 clinical MRSA ST398 isolates.

Antimicrobial agent	Resistant isolates	Resistance rate (%)	Resistance determinants	Determinant-positive isolate	Determinant-positive rate (%)
Penicillin G (PEN)	23/23	100.0%	*mecA*	23/23	100.0%
			*blaZ*	22/23	95.7%
Oxacillin (OXA)	23/23	100.0%	*mecA*	23/23	100.0%
Erythromycin (ERY)	15/23	65.2%	*ermC*	14/23	60.9%
			*ermA*	1/23	4.3%
			*ermB*	1/23	4.3%
Clindamycin (CLI)	15/23	65.2%	*ermC*	14/23	60.9%
			*ermA*	1/23	4.3%
Gentamicin (GEN)	0/23	0.0%	*aacA-aphD*	1/23	4.3%
Rifampin (RIF)	1/23	4.3%	*rpoB*_H481N	1/23	4.3%
Moxifloxacin (MFX)	1/23	4.3%	*grlA*_S80F	1/23	4.3%
			*gyrA*_S84L	1/23	4.3%
Ciprofloxacin (CIP)	1/23	4.3%	*grlA*_S80F/*gyrA*_S84L	1/23	4.3%
Trimethoprim–sulfamethoxazole (SXT)	1/23	4.3%	None detected	0/23	0.0%
Tetracycline (TCY)	1/23	4.3%	*tetL*	1/23	4.3%
Nitrofurantoin (NIT)	0/23	0.0%	None detected	0/23	0.0%
Linezolid (LNZ)	0/23	0.0%	None detected	0/23	0.0%
Vancomycin (VAN)	0/23	0.0%	None detected	0/23	0.0%
Quinupristin/dalfopristin (QDA)	0/23	0.0%	None detected	0/23	0.0%
Tigecycline (TGC)	0/23	0.0%	None detected	0/23	0.0%

The results of antimicrobial susceptibility tests were interpreted according to CLSI M100 criteria. Resistant isolates were those categorized as resistant by phenotypic antimicrobial susceptibility testing; intermediate isolates were not considered resistant. One isolate showed intermediate susceptibility to gentamicin and carried aacA-aphD. Resistance determinants were identified using the Pathogenwatch AMRsearch/PAARSNP pipeline with the curated *S. aureus* AMR library (NCBI taxonomy code 1280; PAARSNP AMR library version 0.0.16 in this analysis). “None detected” indicates that no corresponding determinant was identified by the Pathogenwatch AMR pipeline for that antimicrobial agent.

The genome-based antimicrobial resistance analysis showed that *mecA* was detected in all 23 isolates, supporting the phenotypic resistance to oxacillin and the MRSA classification. The beta-lactamase gene *blaZ* was detected in 22 isolates. Macrolide–lincosamide resistance was the most common non-beta-lactam resistance phenotype: 15 isolates were resistant to erythromycin and 15 were resistant to clindamycin. The major determinant associated with this phenotype was *ermC*, which was detected in 14 isolates, whereas *ermA* and *ermB* were detected in 1 isolate. For clindamycin, *ermC* was detected in 14 isolates, and *ermA* was detected in 1 isolate, corresponding to the 15 clindamycin-resistant isolates.

One isolate showed a broader resistance-associated profile than the remaining isolates. This isolate carried *aacA-aphD*, *rpoB*_H481N, *grlA*_S80F, *gyrA*_S84L, *tetL*, *ermA*, and *ermB*, corresponding to aminoglycoside-, rifampin-, fluoroquinolone-, tetracycline-, and macrolide–lincosamide-associated resistance determinants, respectively. Phenotypically, this isolate showed resistance to rifampin, moxifloxacin, ciprofloxacin, trimethoprim–sulfamethoxazole, erythromycin, clindamycin, and tetracycline, together with intermediate susceptibility to gentamicin. Overall, these results indicate that the clinical MRSA ST398 isolates in this collection uniformly carried *mecA* and were resistant to beta-lactams, while most retained susceptibility to several clinically relevant non-beta-lactam agents. Macrolide–lincosamide resistance was common, and broader multidrug resistance was observed in only one isolate.

### 3.4 Phylogenetic analysis

A total of 1,194 *S. aureus* genomes, including 1,164 PubMLST ST398 genomes, 23 local ST398 genomes from this study, and 7 standard/reference genomes, were initially included in the phylogenetic analysis and phylogenetic dating. The 1,164 PubMLST genomes were annotated in the database and were collected from 30 countries and regions between 1993 and 2023. Among the genomes with available source metadata, most were annotated as animal-associated isolates, followed by human isolates and isolates from other sources. These metadata were used only to describe the composition of the public genomes included in the phylogenetic dataset and were not interpreted as representing the global epidemiological distribution of ST398 (Supplementary Figure S1).

During LSD2-based phylogenetic dating, Newman and MRSA252 were identified as date outliers and excluded, leaving 1,192 genomes in the final time-scaled phylogenetic tree. In this tree, 22 of the 23 local MRSA ST398 isolates from this study clustered within a China-associated clade. In accordance with the available PubMLST metadata, all the genomes within this clade were annotated as isolates from China. Apart from the 22 local clinical isolates, this clade also contained two publicly available ST398 genomes from raw milk products in China collected in 2018 (PubMLST IDs: 42782 and 42783), indicating that closely related genomes have been detected in both clinical and non-human-associated sources in China.

**Figure 4:**
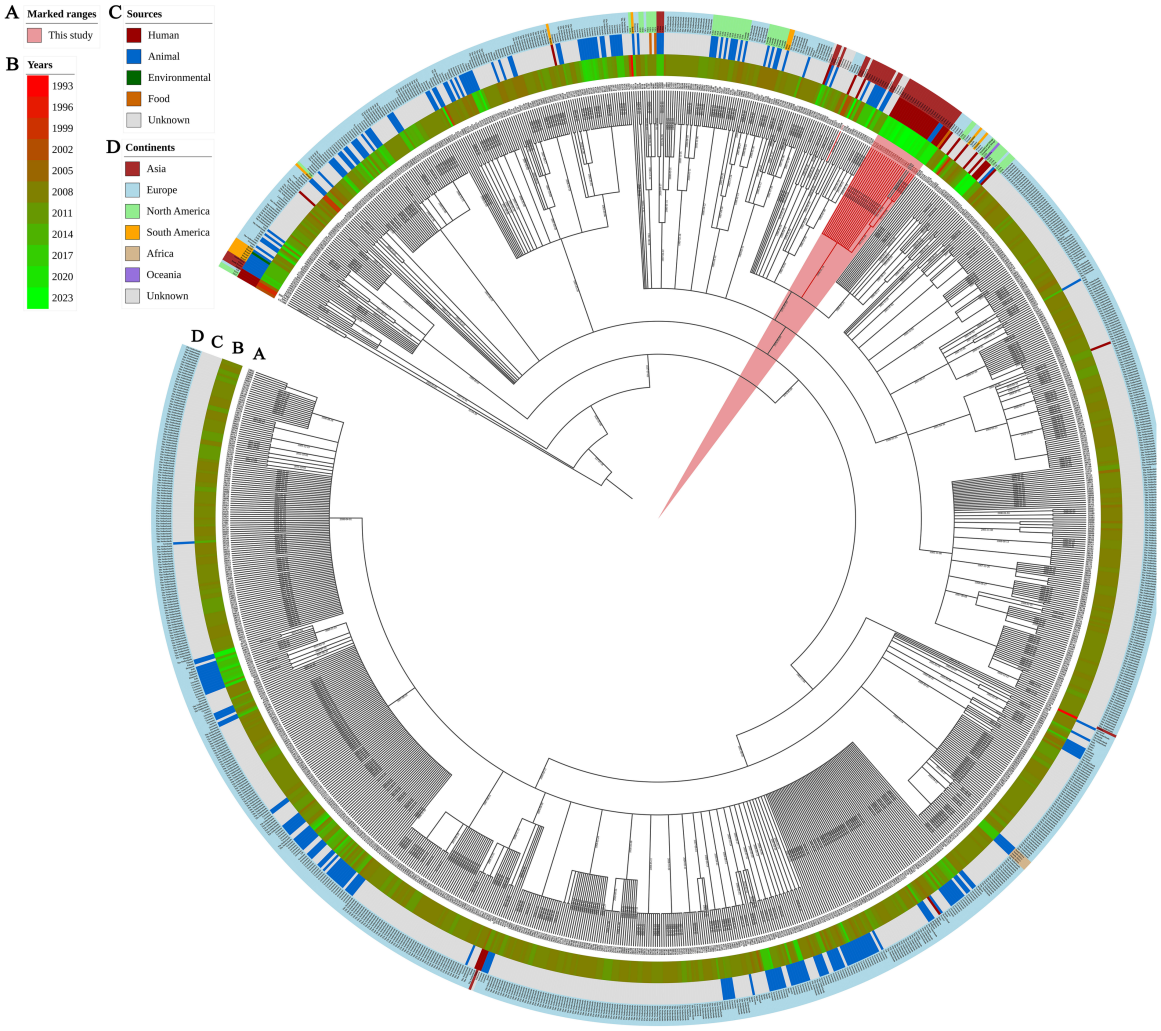
Time-scaled phylogenetic analysis of 1,192 *S. aureus* genomes. The final time-scaled tree included 1,164 PubMLST ST398 genomes, 23 local MRSA ST398 genomes from this study, and 5 standard/reference genomes retained after date-outlier exclusion. Phylogenetic dating was performed using IQ-TREE with least-squares dating implemented in LSD2. **(A)** The red-highlighted clade indicates the China-associated clade containing 22 local clinical isolates from this study and two publicly available ST398 genomes from raw milk products in China. **(B)** The color gradient indicates the annotated sampling years of the genomes from 1993 to 2023. **(C)** Isolation source metadata for the genomes. **(D)** Country/region metadata summarized by continent. The estimated internal node date of the China-associated clade was approximately 2008, with a 95% confidence interval from 2006 to 2013; this estimate was used only to provide the temporal context for the observed clustering pattern.

The temporal signal was assessed with a root-to-tip regression model using the maximum-likelihood tree and sampling dates. The ST398-only dataset showed a detectable but limited temporal signal (R^2^ = 0.0506, *p* < 0.001), and the China-associated clade also showed a detectable temporal signal (R^2^ = 0.2350, *p* = 0.016) (Supplementary Figure S2). Phylogenetic dating estimated that the internal node of this China-associated clade was approximately 2008, with a 95% confidence interval from 2006 to 2013 (Figure 4). All the MRSA isolates within this clade carried *SCCmec* type V but exhibited different *spa* types. This estimate was used to provide the temporal context for the observed clustering pattern and was not interpreted as direct evidence of the transmission direction, independent human adaptation, or a definite transmission chain.

### 3.5 Virulence-associated gene profiles among the ST398 MRSA strains

**Figure 5:**
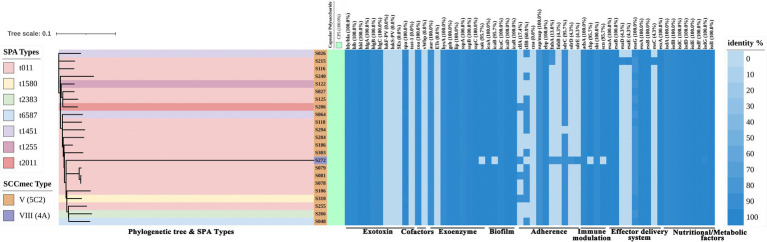
Virulence-associated gene profiles of the 23 clinical MRSA ST398 isolates. The heatmap shows the detection and sequence identity of virulence-associated genes screened against the reference database. Genes are grouped according to functional categories, including exotoxins, cofactors, exoenzymes, biofilm formation-related genes, adherence-related genes, immunomodulatory genes, effector delivery system-related genes, and nutritional/metabolic factors. The phylogenetic tree and molecular typing annotations are shown on the left. The color scale represents sequence identity percentage and should be interpreted as gene detection/identity rather than gene expression, protein activity, or a direct virulence level. SEs: Staphylococcal enterotoxin genes, including the *sea*, *sec*, *seh*, *selk*, *sell* and *selq* genes. ETs: Exfoliative toxin genes, including the *eta*, *etb*, *etd* and *ete* genes.

Virulence-associated gene profiles were analyzed for the 23 clinical MRSA ST398 isolates from this study (Figure 5). A total of 58 virulence-associated genes or gene groups were screened, and detailed detection rates are provided in Supplementary Table S3. Among these genes, 37 were detected in all the isolates, 7 were not detected in any of the isolates, and 14 showed variable distributions among the isolates. Overall, the isolates shared a broad set of conserved virulence-associated genes, while several accessory genes differed among the isolates.

Genes encoding hemolysins or exotoxin-associated factors, including *hly/hla, hlb, hld, hlgA, hlgB, hlgC*, and *spa*, were detected in all the isolates. In contrast, *lukF-PV, lukS-PV*, the screened staphylococcal enterotoxin genes, and *tsst-1* were not detected in any of the isolates. Several genes associated with exoenzymes, biofilm formation, immune modulation, the type VII secretion system, and iron acquisition were also conserved across all the isolates, including *aur, hysA, geh, lip, sspA, sspB, sspC, icaA, icaC, icaD, icaR, adsA, sbi, esaA, esaB, esaG, essA, essB, esxA*, and *isdA–isdI*. All the isolates were assigned to capsular polysaccharide type 5.

The variable genes were mainly accessory virulence-associated genes, particularly genes related to adhesion, immune modulation, and the type VII secretion system. The ST398-VIII-t011 isolate showed a distinct accessory gene profile, lacking *sak, icaB, chp*, and *scn*, but was the only isolate that carried *fnbB, sdrD*, and *sdrE*. Other variable genes included *clfA* detected in 4 isolates (17.4%), *clfB* in 14 isolates (60.9%), *fnbA* in 3 isolates (13.0%), and *sdrC* in 22 isolates (95.7%). The type VII secretion system-related genes *esaD, esaE,* and *essC* were detected in only 1 isolate each (4.3%).

Because many virulence-associated genes were conserved across all or nearly all the isolates, their presence alone was not considered sufficient to explain the differences in the pathogenicity-associated phenotypes. Therefore, these genomic profiles were interpreted as background information and were considered together with the subsequent phenotypic assays rather than as direct evidence of differential virulence.

### 3.6 ST398 MRSA strain growth, biofilm formation and cell adhesion assays

**Figure 6:**
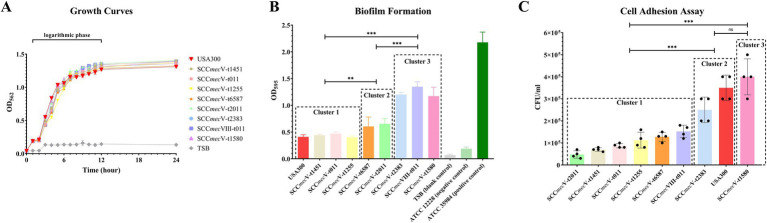
Growth curves, biofilm formation assays, and cell adhesion assays. **(A)** Growth curves of the tested ST398 MRSA lineages and the reference strain USA300. The logarithmic growth phase was observed approximately from the 1st hour to the 12th hour. The data are presented as the means ± SDs from 3 experimental units for each lineage. **(B)** Biofilm formation assay. Ward’s hierarchical clustering analysis based on blank-corrected OD495 values divided the 9 *S. aureus* groups into 3 phenotypic clusters. The data are presented as the means ± SDs from 4 experimental units for each lineage. ** indicates *p* < 0.010, and *** indicates *p* < 0.001. **(C)** Cell adhesion assay. Ward’s hierarchical clustering analysis based on CFU counts divided the 9 *S. aureus* groups into 3 phenotypic clusters. The data are presented as the means ± SDs from 4 experimental units for each lineage. “ns” indicates no statistically significant difference, and *** indicates *p* < 0.001.

In the growth curve analysis, all the tested ST398 MRSA strains and the reference strain USA300 showed broadly comparable growth patterns under the tested conditions. The logarithmic growth phase was observed approximately from the 1st hour to the 12th hour, after which the cultures gradually approached the stationary phase. No marked delay in entry into the logarithmic growth phase was observed among the tested strains (Figure 6A).

In the biofilm formation assay, in accordance with the predefined interpretive criteria, all the tested *S. aureus* strains were classified as weakly positive for biofilm formation. However, quantitative differences in blank-corrected OD495 values were observed among the selected isolates within each lineage. Ward’s hierarchical clustering analysis divided the 9 *S. aureus* groups, including the reference strain USA300, into three phenotypic clusters from low to high biofilm-forming capacity: (1) Cluster 1 (0.362 ± 0.038): USA300, ST398-V-t1451, ST398-V-t011, and ST398-V-t1255; (2) Cluster 2 (0.561 ± 0.135): ST398-V-t6587 and ST398-V-t2011; and (3) Cluster 3 (1.171 ± 0.130): ST398-V-t2383, ST398-VIII-t011, and ST398-V-t1580. Welch’s ANOVA followed by the Games–Howell *post hoc* test revealed significant differences among the clusters (P_1–2_ = 0.010, P_2–3_ < 0.001, and P_1–3_ < 0.001) (Figure 6B).

In the cell adhesion assay, Ward’s hierarchical clustering analysis divided the 9 *S. aureus* groups into three phenotypic clusters according to their adhesion capacity. The quantitative values from low to high were as follows: (1) Cluster 1 ([9.96 ± 4.10] × 10^4^ CFUs/mL): ST398-V-t2011, ST398-V-t1451, ST398-V-t011, ST398-V-t1255, ST398-V-t6587, and ST398-VIII-t011; (2) Cluster 2 ([30.00 ± 7.56] × 10^4^ CFUs/mL): USA300 and ST398-V-t2383; and (3) Cluster 3 ([40.00 ± 8.16] × 10^4^ CFUs/mL): ST398-V-t1580. Welch’s ANOVA followed by the Games–Howell post hoc test showed that Cluster 1 differed significantly from Cluster 2 and Cluster 3 (P_1–2_ < 0.001 and P_1–3_ < 0.001), whereas the difference between Cluster 2 and Cluster 3 was not statistically significant (P_2–3_ = 0.185) (Figure 6C).

Taken together, these lineage-based phenotypic assays with selected isolates indicated that ST398-V-t2383 and ST398-V-t1580 showed relatively higher biofilm formation and/or cell adhesion capacities among the tested ST398 MRSA lineages. However, because the availability of isolates differed among lineages and several lineages were represented by a single isolate, these results should be interpreted as exploratory phenotypic observations rather than definitive lineage-wide conclusions.

### 3.7 Hemolytic assays with the ST398 MRSA strains

**Figure 7:**
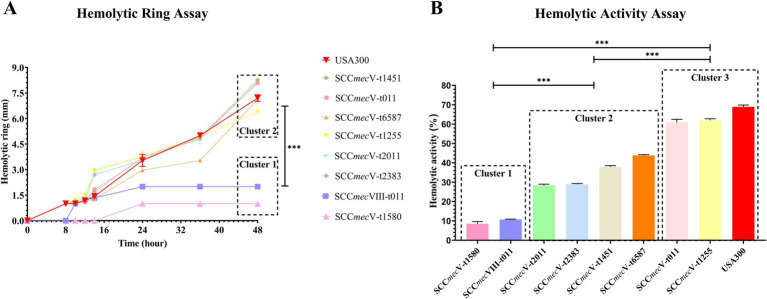
Hemolytic assays. **(A)** Hemolytic ring assay. The hemolytic ring thickness was calculated as the radius of the hemolytic zone minus the radius of the bacterial colony. K-means clustering based on the 48-h hemolytic ring thickness divided the 9 *S. aureus* groups into 2 phenotypic clusters. The data are presented as the means ± SDs from three experimental units for each lineage. **(B)** Hemolytic activity assay. Hemolytic activity was calculated as (OD_test − OD_negative)/(OD_positive − OD_negative) × 100%. K-means clustering, with the number of clusters supported by the silhouette coefficient, divided the 9 *S. aureus* groups into 3 phenotypic clusters. The data are presented as the means ± SDs from three experimental units for each lineage. *** indicates *p* < 0.001.

In the hemolytic ring assay, all the tested lineages produced visible hemolytic rings after 24 h of incubation. At 48 h after inoculation, the hemolytic ring thickness differed among the tested groups. Based on the hemolytic ring thickness at 48 h, k-means clustering divided the nine *S. aureus* groups into two phenotypic clusters: (1) Cluster 1, including ST398-V-t1580 and ST398-VIII-t011; and (2) Cluster 2, including USA300, ST398-V-t1451, ST398-V-t011, ST398-V-t6587, ST398-V-t1255, ST398-V-t2011, and ST398-V-t2383. The hemolytic ring thickness in Cluster 1 was significantly smaller than that in Cluster 2 (1.50 ± 0.55 mm vs. 7.62 ± 0.69 mm, *p* < 0.001) (Figure 7A).

Hemolytic activity assays also revealed marked differences among the tested lineages. K-means clustering, with the number of clusters supported by the silhouette coefficient, divided the 9 *S. aureus* groups into 3 phenotypic clusters from low to high hemolytic activity: (1) Cluster 1, including ST398-V-t1580 and ST398-VIII-t011; (2) Cluster 2, including ST398-V-t2011, ST398-V-t2383, ST398-V-t1451, and ST398-V-t6587; and (3) Cluster 3, including ST398-V-t011, ST398-V-t1255, and USA300. The hemolytic activities of the 3 clusters were 5.00 ± 2.04, 39.86 ± 6.03, and 66.50% ± 5.44%, respectively. Welch’s ANOVA followed by the Games–Howell *post hoc* test revealed significant differences among the clusters (P_1–2_ < 0.001, P_1–3_ < 0.001, and P_2–3_ < 0.001) (Figure 7B).

Taken together, the hemolytic activity of the ST398-V-t1580 and ST398-VIII-t011 lineages was lower than that of the other tested ST398 MRSA lineages under the experimental conditions used in this study.

### 3.8 *In vivo* experiments with the ST398 MRSA strains

**Figure 8:**
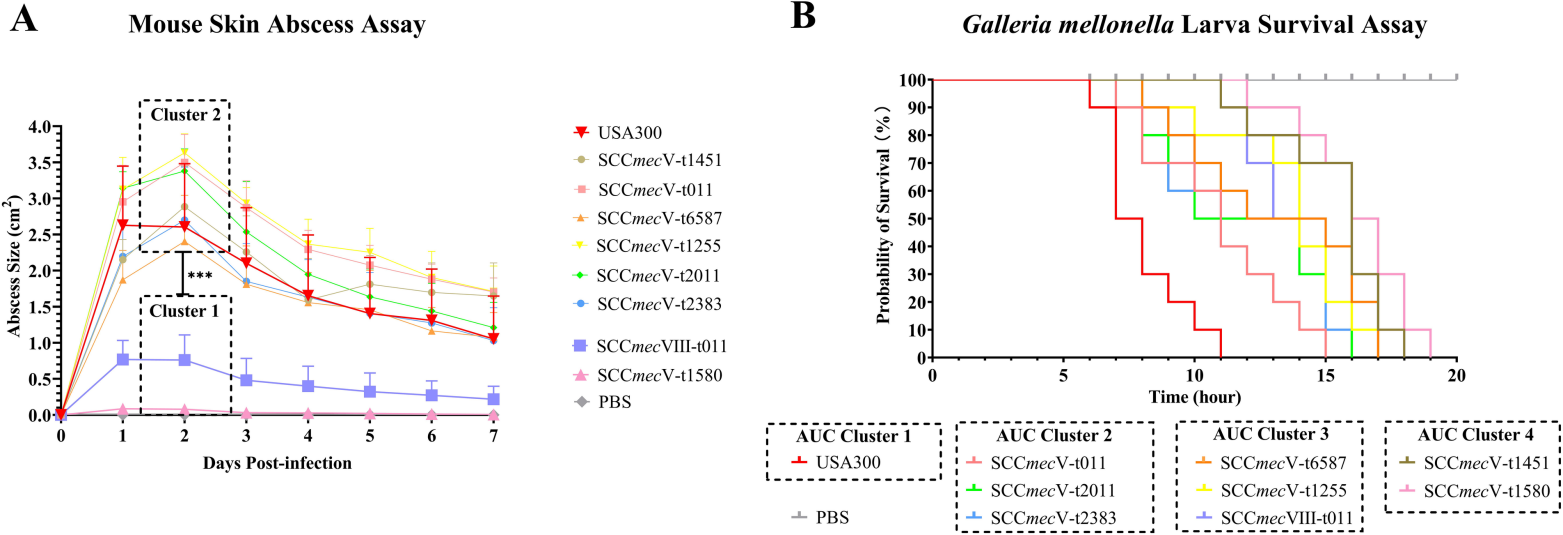
*In vivo* experiments with the ST398 MRSA strains. Each infection group denotes an ST398 lineage or the reference strain USA300. **(A)** Mouse skin abscess assay. Skin abscesses began to form on day 1 after infection and reached their maximum size on day 2. K-means clustering based on the day-2 abscess area divided the 9 *S. aureus* infection groups into 2 phenotypic clusters. The data are presented as the means ± SDs from 5 mice per group. *** indicates p < 0.001 for the intercluster comparison. **(B)** *Galleria mellonella* larval survival assay. Kaplan–Meier survival curves were generated for the 9 *S. aureus* infection groups, with 10 larvae per group. Differences in survival among groups were assessed using the log-rank and Breslow tests. Group-level AUC values were calculated to summarize the overall survival profile of each lineage group or the USA300 reference group, and AUC-based clusters are shown as descriptive phenotypic groupings. The PBS group was included as a negative control and was not included in the AUC-based clustering of the *S. aureus* infection groups.

In the mouse skin abscess assay, mice inoculated with *S. aureus* experienced transient body weight loss on the first day after infection but then gradually recovered (Supplementary Figure S3). Skin abscesses began to form on the first day after infection and reached their maximum size on the second day. Thereafter, the abscesses gradually healed, and the lesion areas decreased. Based on the abscess area measured on day 2 after infection, k-means clustering, with the number of clusters supported by the silhouette coefficient, divided the 9 *S. aureus* groups into 2 phenotypic clusters: (1) Cluster 1, including ST398-V-t1580 and ST398-VIII-t011; and (2) Cluster 2, including USA300, ST398-V-t1451, ST398-V-t011, ST398-V-t6587, ST398-V-t1255, ST398-V-t2011, and ST398-V-t2383. The intercluster comparison revealed that the abscess area in Cluster 1 was significantly smaller than that in Cluster 2 (0.43 ± 0.42 cm^2^ vs. 3.01 ± 0.70 cm^2^, *p* < 0.001) (Figure 8A).

In the *Galleria mellonella* larval survival assay, all infected larvae died within 20 h, whereas the PBS control group showed no mortality during the observation period. The Kaplan–Meier survival analysis showed significant differences among the survival curves of the 9 *S. aureus* infection groups, as assessed using the log-rank test and Breslow test (Supplementary Table S4). Among the tested lineages, USA300 caused the most rapid larval death, whereas ST398-V-t1451 and ST398-V-t1580 resulted in relatively slower death.

The area under the survival curve (AUC) was calculated for each lineage group to further summarize the overall survival profiles. Lower AUC values indicate faster larval death and greater pathogenicity-associated readouts in this model. Based on group-level AUC values, exploratory k-means clustering grouped the lineages into four descriptive phenotypic categories: (1) AUC Cluster 1, USA300, with an AUC of 7.250; (2) AUC Cluster 2, ST398-V-t011, ST398-V-t2011, and ST398-V-t2383, with an AUC of 11.000 ± 0.541; (3) AUC Cluster 3, ST398-V-t6587, ST398-V-t1255, and ST398-VIII-t011, with an AUC of 13.017 ± 0.382; and (4) AUC Cluster 4, ST398-V-t1451 and ST398-V-t1580, with an AUC of 15.250 ± 0.636 (Figure 8B). Because each lineage group contributed one group-level AUC value, AUC-based clustering was used only as a descriptive exploratory analysis and was not subjected to an inferential intercluster comparison.

Taken together, these *in vivo* models showed heterogeneous pathogenicity-associated phenotypes among the tested ST398 MRSA lineages. ST398-V-t011 belonged to a higher-pathogenicity-associated group in the *Galleria mellonella* model and to the larger-abscess cluster in the mouse skin abscess model, despite showing relatively weak biofilm formation and adhesion capacities. However, because the isolate availability differed among lineages and several lineages were represented by a single isolate, these findings should be interpreted as lineage-based exploratory observations under the tested conditions rather than definitive lineage-wide conclusions.

## 4 Discussion

In this study, we characterized 23 nonduplicate clinical MRSA ST398 isolates identified among 305 nonduplicate MRSA isolates from a women and children’s hospital in Southwest China. By integrating antimicrobial susceptibility testing, whole-genome sequencing, phylogenetic analysis, virulence-associated gene screening, and lineage-based phenotypic assays using selected clinical isolates, this study provides regional data on the molecular and pathogenicity-associated features of clinical MRSA ST398 isolates. The predominant lineage was ST398-V-t011, accompanied by a high proportion of *SCCmec* type V and spa type t011. All the isolates were *mecA*-positive and phenotypically resistant to beta-lactams, whereas most of the isolates retained susceptibility to several non-beta-lactam agents, with macrolide–lincosamide resistance being the most common non-beta-lactam resistance phenotype. The phylogenetic analysis placed most local isolates within a China-associated clade, and lineage-based phenotypic assays using the selected isolates revealed heterogeneous biofilm formation, cell adhesion, hemolytic activity, and *in vivo* pathogenicity-associated readouts among the tested ST398 lineages.

ST398 was first recognized largely in the context of livestock-associated *S. aureus* epidemiology. An early report by Armand-Lefevre et al. showed clonal relatedness among *S. aureus* isolates from pigs, pig farmers, and human controls, drawing attention to ST398 as a lineage with potential links to livestock exposure (43). Subsequent studies found LA-MRSA ST398 in pigs, dairy cattle, poultry products, meat products, and individuals with occupational or environmental contact with livestock, supporting the importance of livestock and food-chain reservoirs in the epidemiology of this lineage (44–47). Nevertheless, ST398 should not be regarded as synonymous with LA-MRSA. Accumulating evidence indicates that ST398 includes both livestock-associated and human-associated lineages and that genetic adaptation, mobile genetic elements, and host-associated genomic backgrounds may influence its epidemiological behavior in different settings (48, 49). ST398 has been increasingly reported in clinical infections in humans worldwide, particularly in Europe and China, but many previous studies have focused on MSSA or CC398 rather than MRSA ST398 specifically (15, 49–58). Therefore, when MRSA ST398 is detected in human clinical infections, its epidemiological interpretation should consider both possible livestock-related origins and human-associated dissemination while avoiding the assumption that livestock contact is always identifiable or that phylogenetic relatedness alone can establish a specific transmission route. Large-scale molecular epidemiological studies focusing specifically on clinical MRSA ST398 remain limited, especially in China.

In the present study, 23 MRSA ST398 isolates were identified among 305 nonduplicate clinical MRSA isolates, corresponding to a detection rate of 7.5%. Most patients were infants or young children, consistent with the clinical setting of a women and children’s hospital. Pneumonia was the most common clinical diagnosis, accounting for 19 of the 23 cases (82.6%), and sputum was the most frequent specimen type, accounting for 17 isolates (73.9%). All included isolates were considered clinically relevant based on clinical manifestations, imaging findings, laboratory test results, microbiological culture results, and corresponding ICD diagnostic criteria, rather than culture positivity alone. No documented history of livestock contact within 1 month before disease onset was recorded in the available clinical information. However, this result should not be interpreted as excluding indirect, environmental, food chain, or undocumented exposure. At the molecular level, ST398-V-t011 was the predominant lineage, accounting for 15 isolates (65.2%), whereas spa type t011 and *SCCmec* type V were detected in 16 isolates (69.6%) and 22 isolates (95.7%), respectively.

The antimicrobial resistance profile of MRSA ST398 should be interpreted in the broader context of ST398 ecology and host-associated evolution. Previous genomic studies have suggested that CC398 originated from human-associated MSSA backgrounds and subsequently acquired livestock-associated resistance traits, including *SCCmec*-mediated methicillin resistance and tetracycline resistance, after expansion in food-producing animals (59). Consistent with this ecological pattern, studies of livestock-, slaughterhouse-, and food chain-associated MRSA ST398 have frequently reported multidrug resistance, with resistance to tetracyclines, macrolides, lincosamides, aminoglycosides, and fluoroquinolones being variably detected across settings (60, 61). In this context, the resistance pattern observed in our clinical isolates was relatively focused: all the isolates were *mecA-*positive and resistant to penicillin G and oxacillin, whereas all the isolates remained susceptible to vancomycin and linezolid, and only one isolate showed a broader multidrug resistance profile. The most prominent non-beta-lactam resistance phenotype was macrolide–lincosamide resistance, with erythromycin and clindamycin resistance each detected in 15 isolates (65.2%), which were associated mainly with *ermC*. These findings differ from those presented in many livestock-associated ST398 reports in which tetracycline resistance is often prominent. However, the findings are broadly consistent with those presented in recent reports of Chinese pig-associated MRSA ST398 showing frequent beta-lactam and macrolide–lincosamide resistance in t011-*SCCmec* V backgrounds (61). More generally, these comparisons emphasize that MRSA ST398 resistance profiles are not fixed lineage properties but are shaped by host reservoirs, antimicrobial selection pressure, and mobile genetic elements. Therefore, phenotypic AST should be interpreted together with resistance determinants. Recent borderline oxacillin-resistant *S. aureus* (BORSA) studies have highlighted the diagnostic ambiguity and therapeutic challenges associated with *β*-lactam resistance phenotypes, particularly when borderline oxacillin resistance occurs in the absence of canonical mec-mediated resistance determinants (62–64). In the present study, the concordance between phenotypic oxacillin resistance and *mecA* detection supports the classification of these isolates as *mecA*-positive MRSA rather than BORSA, whereas the results of the combined AST and genomic resistance determinant analyses provide a more clinically informative AMR profile for MRSA ST398 in this cohort.

The phylogenetic results should also be interpreted cautiously. In the final time-scaled tree, 22 of the 23 local MRSA ST398 isolates clustered within a China-associated clade, and all the genomes in this clade with available metadata were annotated as isolates from China. This clustering suggests that the local clinical isolates were genetically close to a subset of China-associated ST398 genomes rather than being randomly distributed across the global ST398 phylogeny. Notably, the same clade also included two publicly available ST398 genomes from raw milk products in China, indicating that closely related ST398 genomes have been detected in both clinical and nonhuman-associated sources. However, this observation does not establish a direct epidemiological link between these sources. LSD2-based dating estimated the internal node of this clade to be approximately 2008, with a 95% confidence interval from 2006 to 2013. Because the temporal signal was detectable but limited and because publicly available PubMLST genomes are subject to uneven geographic, host-source, and sampling-time representation, these data should be considered a temporal context for the observed clustering pattern rather than definitive evidence of when or where this lineage emerged. Similarly, the absence of documented livestock contact in the clinical records does not exclude indirect, food chain, environmental, or undocumented exposure. Therefore, the phylogenetic findings support the presence of a China-associated genomic cluster containing most local isolates, but they do not allow drawing of firm conclusions regarding the transmission direction, independent human adaptation, or a definite transmission chain.

Virulence-associated gene profiles provide a useful genomic background for interpreting the pathogenicity-associated phenotypes of these MRSA ST398 isolates, but they should not be overinterpreted as direct indicators of virulence. In this study, 37 of the 58 screened virulence-associated genes or gene groups, including genes encoding hemolysins, exoenzymes, biofilm-associated factors, immunomodulatory factors, type VII secretion system components, and iron-acquisition factors, were detected in all the isolates. This high level of conservation indicates that the presence of these genes alone is unlikely to explain the phenotypic differences observed among the tested ST398 lineages. Conversely, 14 genes showed a variable distribution and mainly included accessory genes related to adhesion, immune modulation, and the type VII secretion system. These variable genes may provide clues to genomic heterogeneity among MRSA ST398 isolates, but their biological effects cannot be inferred from gene detection alone. Differences in gene expression, regulatory networks, protein production, toxin activity, and host–pathogen interactions may all influence the observed phenotypes. Therefore, virulence-associated gene profiles in this study should be interpreted as a framework for generating mechanistic hypotheses rather than as direct evidence of lineage-specific virulence.

The phenotypic assays further showed that pathogenicity-associated traits were not uniform across the tested MRSA ST398 lineages and did not follow simple gene-presence or lineage-prevalence patterns. The ST398-V-t1580 and ST398-V-t2383 lineage groups presented relatively increased biofilm formation and/or cell adhesion capacities, suggesting increased readouts in assays related to surface attachment and epithelial cell interactions under the tested conditions. However, these phenotypes were not accompanied by higher hemolytic activity or consistent increases in pathogenicity-associated readouts in the *in vivo* models. Conversely, the ST398-V-t011 lineage group showed relatively weak biofilm formation and adhesion capacities but belonged to a cluster with higher hemolytic activity and showed higher pathogenicity-associated readouts under the tested conditions in both the mouse skin abscess and *Galleria mellonella* models. This apparent discordance is biologically plausible because biofilm formation, epithelial adhesion, hemolysis, abscess formation, and larval killing reflect different aspects of bacterial fitness and host–pathogen interactions rather than a single unified virulence scale. Therefore, the phenotype of ST398-V-t011 should be interpreted as a stronger response in selected pathogenicity-associated assays, not as definitive evidence that this lineage is overall more virulent. These findings also suggest that the observed phenotypic heterogeneity may be influenced by regulatory activity, toxin expression, the metabolic state, and host-context-dependent interactions, which cannot be resolved by virulence-associated gene detection alone. Thus, the combined genomic and phenotypic data highlight the value of using multiple complementary assays to evaluate MRSA ST398 while also underscoring the need for mechanistic validation before specific genetic backgrounds are linked to lineage-wide pathogenic behavior.

To our knowledge, this study provides one of the few integrated descriptions of clinical MRSA ST398 isolates from Southwest China, particularly from a women and children’s hospital. Its main value lies in combining clinical data, whole-genome-based molecular typing, antimicrobial susceptibility and resistance determinant profiling, phylogenetic analysis, virulence-associated gene screening, and lineage-based phenotypic assays using selected clinical isolates within the same isolate collection. Compared with studies focusing mainly on livestock-associated ST398 or MSSA CC398, this work adds regional information on *mecA*-positive clinical MRSA ST398 isolates and shows that ST398-V-t011 was the dominant lineage in this cohort. The phylogenetic analysis further placed most local isolates within a China-associated clade and provided a temporal estimate for this clustering pattern. However, these findings were interpreted as a genomic context rather than as proof of the transmission direction or independent spread. In addition, the results of the phenotypic assays showed that the tested ST398 lineages exhibited distinct pathogenicity-associated profiles across adhesion, biofilm formation, hemolysis, mouse skin abscess, and *Galleria mellonella* survival models. These findings do not establish lineage-wide virulence mechanisms, but they provide a useful basis for generating hypotheses and guiding future multicenter genomic and functional studies of MRSA ST398.

This study has several limitations. First, although the 23 MRSA ST398 isolates were identified through the continuous screening of 1,247 clinical *S. aureus* isolates and 305 nonduplicate MRSA isolates over a four-year period at a large women and children’s hospital in Southwest China, the absolute number of ST398 isolates remained limited. Therefore, MRSA ST398 detection in this clinical collection is epidemiologically meaningful for regional surveillance, but the statistical power for lineage-level comparisons and the generalizability of the findings remain constrained. In addition, because this study was conducted in a specialized women and children’s hospital, the patient population was skewed toward infants, children, and women, and respiratory specimens accounted for a large proportion of the isolates. Thus, the observed clinical spectrum, specimen distribution, and molecular lineage composition may not fully represent the presence of MRSA ST398 in general hospitals, adult populations, or other regions. Second, livestock exposure was assessed retrospectively from the available clinical records. Although no documented livestock contact was recorded, indirect exposure through food chain, environmental, household, or unrecognized contact could not be excluded. Because animal, food, environmental, and household samples were not collected, the sources and transmission routes of these isolates could not be determined. Third, the phylogenetic analysis incorporated publicly available PubMLST genomes with heterogeneous geographic origins, host sources, sampling times, and metadata completeness. Although most local isolates clustered within a China-associated clade and LSD2-based dating provided a temporal estimate for this clustering pattern, these results should be interpreted in a genomic and temporal context rather than as evidence of the transmission direction, independent human adaptation, or a definite transmission chain. Fourth, although the phenotypic assays were performed using selected clinical isolates within each lineage, the availability of the isolates differed markedly among lineages. For lineages with sufficient available isolates, nonduplicate isolates were randomly selected whenever possible; for lineages represented by two or one isolate, the available isolates had to be reused as evenly as possible across repeated assays, mice, or larvae. Therefore, the observed differences in biofilm formation, cell adhesion, hemolysis, mouse skin abscess formation, and *Galleria mellonella* survival may reflect both lineage-associated tendencies and isolate-specific variation. These findings should be interpreted as exploratory pathogenicity-associated observations under the tested conditions rather than definitive evidence of genotype-wide or lineage-wide differences in virulence. Finally, the screening of virulence-associated genes was based on gene detection and sequence identity and did not involve assessments of gene expression, protein production, toxin activity, regulatory variation, or functional effects. Future multicenter studies with larger isolate collections, broader source sampling, and transcriptomic, proteomic, and genetic validation are needed to clarify the epidemiology, transmission routes, and mechanisms underlying pathogenicity-associated variation in MRSA ST398.

## 5 Conclusion

In summary, this study identified 23 clinical MRSA ST398 isolates through continuous screening of clinical *S. aureus* isolates from a women and children’s hospital in Southwest China, with ST398 accounting for 7.5% of the nonduplicate clinical MRSA isolates in this collection. ST398-V-t011 was the dominant lineage, and all the isolates were *mecA*-positive and phenotypically resistant to beta-lactams, while most retained susceptibility to several clinically relevant non-beta-lactam agents. The phylogenetic analysis placed most local isolates within a China-associated clade and provided a temporal estimate for this clustering pattern. However, these findings should be interpreted in a genomic and temporal context rather than as direct evidence of the transmission direction, independent human adaptation, or a definite transmission chain. Lineage-based phenotypic assays using the selected clinical isolates revealed heterogeneous pathogenicity-associated phenotypes across biofilm formation, cell adhesion, hemolysis, mouse skin abscess, and *Galleria mellonella* survival models. These findings expand the regional knowledge of clinical MRSA ST398 in Southwest China and support the need for continued genomic surveillance, antimicrobial resistance monitoring, and larger multicenter studies to clarify its epidemiology, transmission routes, and pathogenicity-associated mechanisms.

## Data Availability

The datasets presented in this study can be found in online repositories. The names of the repository/repositories and accession number(s) can be found at https://www.ncbi.nlm.nih.gov/genbank/, PRJNA1090615.

## Footnotes

1
https://github.com/pathogenwatch-oss/amr-search

2
https://pathogen.watch/

3
https://cge.food.dtu.dk/services/spaTyper

4
https://pubmlst.org/

## Supplementary material

SUPPLEMENTARY FIGURE S1 Metadata distribution of PubMLST ST398 genomes included in the phylogenetic analysis. **(A)** Country or region distribution of the 1,164 PubMLST ST398 genomes with available genome sequences and sampling years. **(B)** Continent-level distribution of these PubMLST genomes. **(C)** Source-category distribution according to the metadata recorded in PubMLST. These metadata were used only to describe the composition of the public genome dataset and should not be interpreted as reflecting the global prevalence, source distribution, or transmission pattern of ST398 because PubMLST records are derived from heterogeneous submissions with uneven geographic representation and incomplete source annotations.

SUPPLEMENTARY FIGURE S2 Temporal signal assessment for phylogenetic dating. **(A)** Root-to-tip regression for the ST398-only dataset after excluding the seven standard/reference genomes. **(B)** Root-to-tip regression for the China-associated target clade containing 22 local isolates from this study and PubMLST genomes 42782 and 42783. Root-to-tip distances were calculated from the maximum-likelihood tree and plotted against sampling dates. The results showed detectable but limited temporal signal, supporting cautious interpretation of the LSD2-based dating estimates.

SUPPLEMENTARY FIGURE S3 Mouse body-weight changes after infection in the skin abscess model. Body-weight changes of mice infected with selected MRSA ST398 lineage-representative isolates in the mouse skin abscess model. Body weight was monitored from day 0 to day 7 post-infection. Data are presented as the mean ± SD for each lineage group. These data were used as supportive monitoring of animal condition during the skin abscess experiment rather than as a primary endpoint for lineage-level pathogenicity inference.

SUPPLEMENTARY TABLE S1 Genome assembly quality metrics of 23 clinical MRSA ST398 isolates.

SUPPLEMENTARY TABLE S2 Isolate allocation scheme for lineage-based phenotypic assays.

SUPPLEMENTARY TABLE S3 Detection rates of virulence-associated genes among 23 clinical MRSA ST398 isolates.

SUPPLEMENTARY TABLE S4 Pairwise comparison of Kaplan–Meier survival curves using log-rank and Breslow tests.
