# Supplementary figures and images for: Molecular characteristics and pathogenicity-associated phenotypes of methicillin-resistant Staphylococcus aureus sequence type 398 clinical isolates from a women and children’s hospital in Southwest China
Source: Front Public Health. 2026 Jul 6;14:1882026. doi: 10.3389/fpubh.2026.1882026 (PMC13381705; doi:10.3389/fpubh.2026.1882026)

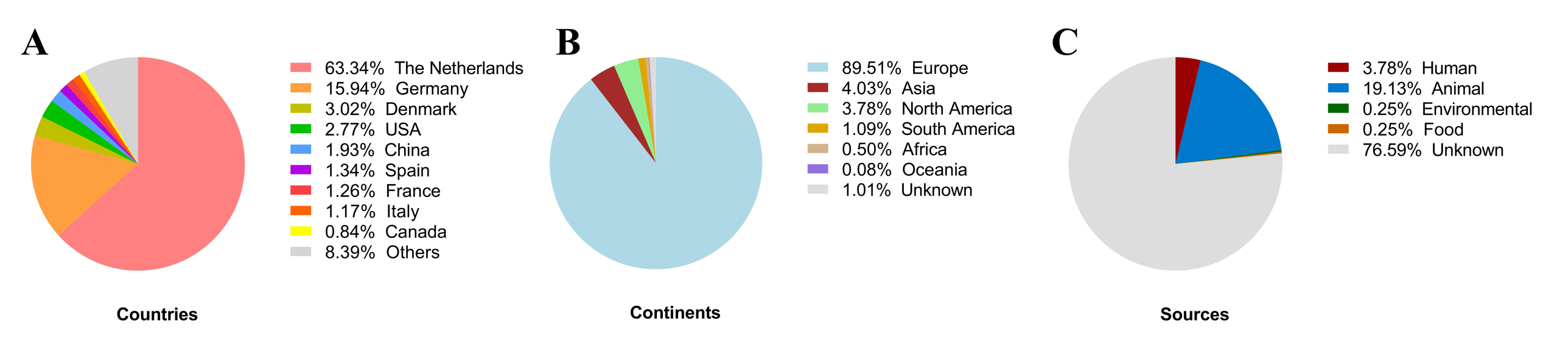

Supplement: SUPPLEMENTARY FIGURE S1 — Metadata distribution of PubMLST ST398 genomes included in the phylogenetic analysis. (A) Country or region distribution of the 1,164 PubMLST ST398 genomes with available genome sequences and sampling years. (B) Continent-level distribution of these PubMLST genomes. (C) Source-category distribution according to the metadata recorded in PubMLST. These metadata were used only to describe the composition of the public genome dataset and should not be interpreted as reflecting the global prevalence, source distribution, or transmission pattern of ST398 because PubMLST records are derived from heterogeneous submissions with uneven geographic representation and incomplete source annotations. [file Image_1.JPEG]

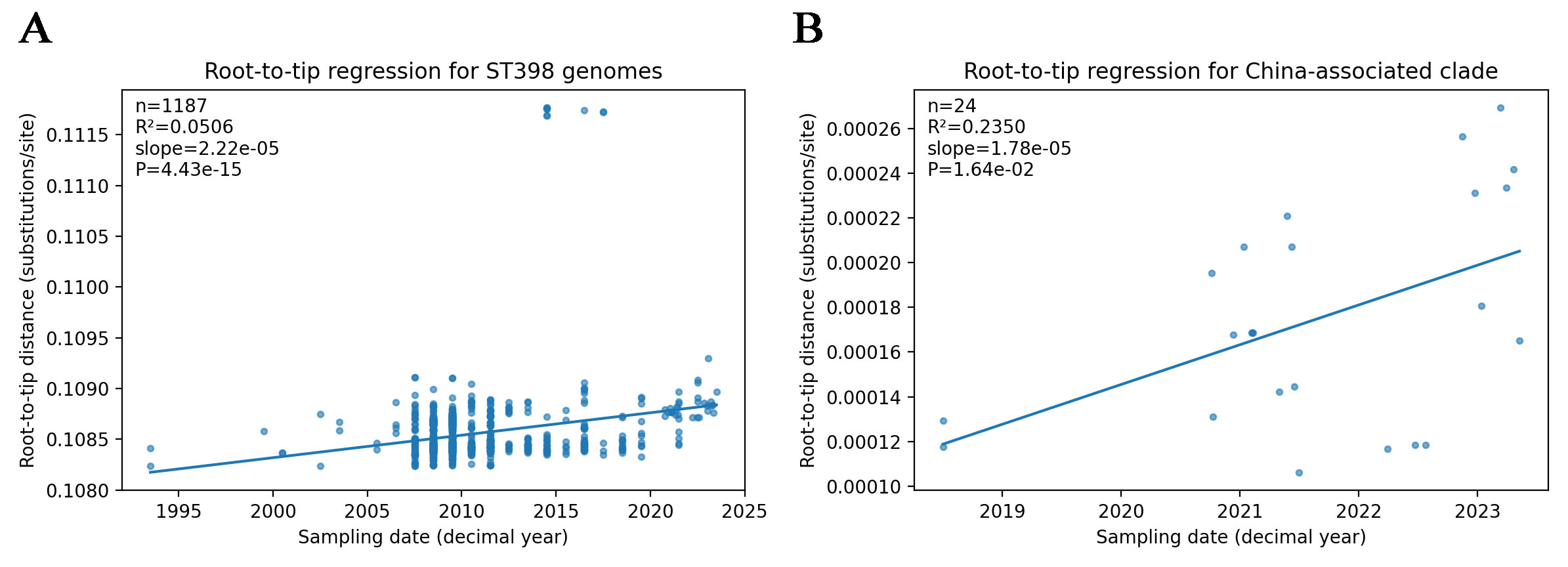

Supplement: SUPPLEMENTARY FIGURE S2 — Temporal signal assessment for phylogenetic dating. (A) Root-to-tip regression for the ST398-only dataset after excluding the seven standard/reference genomes. (B) Root-to-tip regression for the China-associated target clade containing 22 local isolates from this study and PubMLST genomes 42782 and 42783. Root-to-tip distances were calculated from the maximum-likelihood tree and plotted against sampling dates. The results showed detectable but limited temporal signal, supporting cautious interpretation of the LSD2-based dating estimates. [file Image_2.JPEG]
